# Bioinformatics study of biotin carboxylase B-subdomain isolated from *Lactococcus lactis* subsp. *lactis* (Lac3)

**DOI:** 10.5114/bta/207712

**Published:** 2025-08-26

**Authors:** Rafika Dwi Cahyani, Apon Zaenal Mustopa, Rifqiyah Nur Umami, Arwansyah Arwansyah, Setyanto Tri Wahyudi

**Affiliations:** 1 Program of Biotechnology, IPB University, Bogor, Indonesia; 2 Research Center for Genetic Engineering, National Research and Innovation Agency (BRIN), Bogor, Indonesia; 3 Department of Chemistry Education, Faculty of Teacher Training and Education, Tadulako University, Palu, Indonesia; 4 Department of Physics, IPB University, Bogor, Indonesia; 5 Tropical Biopharmaca Research Center, IPB University, Bogor, Indonesia

**Keywords:** whole genome sequencing (WGS), molecular docking, molecular dynamics, *Lactococcus lactis* subsp. *lactis* (Lac3), biotin carboxylase B-subdomain, ATP-grasp superfamily

## Abstract

**Background:**

The increasing threat of antibiotic-resistant bacteria is a significant global health concern, with millions of people worldwide infected with these resistant strains each year. This study aims to conduct a bioinformatics analysis to investigate the biotin carboxylase (BC) B-subdomain from *Lactococcus lactis* subsp. *lactis* (Lac3) (accession number NZ_JAGRPZ010000035.1) as a potential target for the identification and development of novel antibiotics. Lac3 was isolated from one of the Indonesian traditional probiotics called dadih, and its whole-genome sequence analysis was revealed in a previous study.

**Materials and methods:**

Whole-genome sequencing data of Lac3, generated using the Illumina MiSeq sequencer (Novogene Co., Ltd.), were used to analyze gene clusters with AntiSMASH. Molecular docking (PyRx Virtual Screening Tool; AutoDock Vina) and molecular dynamics simulations (CPPTRAJ software) were performed to elucidate the potential binding sites of the BC B-subdomain and compare them with the BC domain from a *L. lactis* reference strain (accession number KLK97304). The 3D structure of the BC B-subdomain was predicted using AlphaFold2. Visualization of the simulated protein–ligand complex conformations was conducted using PyMOL v2.3 software.

**Results:**

Bioinformatics analysis showed that the BC B-subdomain gene was located in the β-lactone gene cluster on contig 7.1 and consisted of 32.1% α-helix, 37.6% β-strand, and 24.8% random coil. Physicochemical analysis indicated that the BC B-subdomain protein exhibited a high degree of solubility. The BC B-subdomain shared similarities with the ATP-grasp domain of the BC domain from the reference strain, particularly in amino acid residues involved in ATP binding (His207, Gln231, Asn234, and Glu274). Molecular docking analysis demonstrated that the BC B-subdomain–ATP complex (–6.1 kcal/mol) was comparable to the BC domain–ATP complex (–8.8 kcal/mol). This was supported by molecular dynamics simulations, which indicated that the complex models remained stable throughout the simulations, based on several validation parameters, including RMSD, RMSF, Rg, and SASA. Furthermore, ionic interactions with the phosphate group’s amino acid residues – critical for ATP binding and function within ATP-grasp enzymes – were observed in both the BC B-subdomain (His207 and Lys236) and the BC domain (Lys236 and Arg290).

**Conclusions:**

These findings suggest that the BC B-subdomain could serve as a potential target for fragment-based drug discovery and may provide a reference for developing novel BC inhibitors with potent antibacterial activity by targeting ATP binding, possibly through its phosphate group binding sites. However, further analysis is needed to support the development of innovative antibacterial treatments in the future.

## Introduction

The increasing prevalence of antibiotic-resistant bacteria poses a significant global health threat, with millions of people suffering from these infections each year. Gram-negative bacteria such as *Klebsiella pneumoniae, Acinetobacter baumannii, Pseudomonas aeruginosa*, and *Enterobacter* – members of the ESKAPE pathogen group – are major causes of hospital-acquired infections. These bacteria are notorious for their ability to evade current antibiotics (Renner et al. 2017). Although some novel antibiotics have been introduced recently, they remain insufficient to meet the current demand, and more potent antibiotics are urgently needed to combat these highly virulent pathogens.

Lactic acid bacteria (LAB) are widely used as starter cultures in the production of probiotics. LAB produces peptides and amino acids with various biological functions, including angiotensin-converting enzyme inhibitors (Nielsen et al. 2009), immune system modulators (Coste et al. 1992), and antioxidants (Peńa-Ramos et al. 2004). Probiotics, therefore, play a role in the body’s defense regulation and can modulate immune responses to oxidative stress, ultimately contributing to the prevention of various diseases, including digestive disorders and cancers (Yahfoufi et al. 2018).

A fermented buffalo milk product called *dadih* is one of Indonesia’s traditional probiotics and contains indigenous LAB such as *Lactobacillus plantarum, Lactobacillus* sp., *Lactococcus* sp., and *Leuconostoc* sp. (Surono and Hosono 2000). *Lactococcus lactis* subsp. *lactis* (Lac3) (accession number NZ_JAGRPZ010000035.1), isolated from *dadih*, has been reported to possess antioxidant activity, with 64.2% inhibition of DPPH (2,2-diphenyl-1-picrylhydrazyl) radicals (Hasim et al. 2017). Recently, Sylvere and colleagues reported wholegenome sequencing (WGS) analysis and revealed beneficial characteristics of Lac3 as a probiotic strain (Sylvere et al. 2023). In addition, our group reported that Lac3 produces secondary metabolites capable of inhibiting cyclooxygenase-2 activity through in silico analysis. These findings suggest that bioactive compounds from Lac3 has the potential to serve as antiinflammatory agents by targeting prostaglandin biosynthesis (Cahyani et al. 2023).

LAB also synthesizes a range of primary metabolites, including pyruvate carboxylase (PC), a large, complex, biotin-dependent carboxylase enzyme. PC plays a pivotal role in various metabolic processes across diverse organisms, particularly in the formation of oxaloacetate (Jitrapakdee et al. 2008). Its unique structure and catalytic activity are essential for carbon metabolism and the virulence of certain pathogens. While both acetyl-CoA and MgTNP-ATP influence PC activity, they do so through distinct mechanisms (Jitrapakdee and Wallace 1999). The genome sequence of PC in *L. lactis* shares 72% similarity with *Enterococcus faecalis* and 63% similarity with *Listeria monocytogenes* (Choi et al. 2017). The PC enzyme consists of multidomain monomers with three functional domains: biotin carboxyl carrier protein (BCCP), biotin carboxylase (BC), and carboxyl transferase (CT). A previous study suggested that the catalytic activity of PC in *Staphylococcus aureus* is influenced by dimerization of the BC domain (Yu et al. 2013). Another study revealed that the crystal structure of the BCCP–BC complex in *Escherichia coli* forms a unique biotin-dependent carboxylase binding site, highlighting its potential as a target for the development of new antibacterial agents (Broussard et al. 2013).

The BC domain is an enzyme belonging to the ATPgrasp superfamily (Škedelj et al. 2011; Brylinski and Waldrop 2014) and consists of subdomains A, B, and C, which are also classified as biotin-dependent carboxylase enzymes (Waldrop et al. 1994). The BC B-subdomain is composed of two α-helices and three β-sheets, with a glycine-loop region at residues 160–166 that plays a crucial role in adenosine triphosphate (ATP) binding. As part of the ATP-grasp superfamily, the BC B-subdomain is involved in *de novo* purine biosynthesis, fatty acid synthesis, gluconeogenesis, and cellular metabolism. During catalysis, the BC B-subdomain dimerizes to close the active site (Thoden et al. 2000). It is known that the ATP binding site in ATP-grasp enzymes is structurally distinct from that of other ATP-binding enzymes, indicating that the ATP-grasp site may serve as a promising target for drug design (Škedelj et al. 2011). Furthermore, the discovery of both ATP-competitive and non-competitive inhibitors for ATP-grasp enzymes highlights the opportunity to explore allosteric mechanisms regulating their function (Fawaz et al. 2011).

Fragment-based drug discovery (FBDD) is a powerful approach for identifying and developing new therapeutics. It involves screening a library of small, simple molecules known as fragments, which are significantly smaller than conventional drug compounds. FBDD has been applied to identify fragments that bind to biotin carboxylase (BC) (Craft and Waldrop 2022). By expanding and refining the binding sites of the BC domain, this strategy provides a new reference for designing novel BC inhibitors with potent antibacterial activity. This underscores the potential of FBDD to accelerate drug discovery and enable the development of groundbreaking treatments for a variety of diseases. In this study, the identification and isolation of the BC B-subdomain from the Lac3 genome was followed by a bioinformatics analysis – including physicochemical characterization, molecular docking, and molecular dynamics simulations – to investigate its ATP binding sites as potential targets for drug development.

## Materials and Methods

### Gene cluster analysis

WGS data of Lac3, generated using the Illumina MiSeq sequencer (Novogene Co., Ltd.), were used to analyze gene clusters encoding secondary and primary metabolites. To ensure data reliability, the quality of the sequencing reads was assessed using FastQC (version 0.11.8) on the UseGalaxy web server (version 0.72+galaxy) (Clabaut et al. 2019). *De novo* assembly was then performed to obtain contigs using the Unicycler assembler algorithm, integrated within the PATRIC (PathoSystems Resource Integration Center) platform (Wick et al. 2017). This integration provided access to QUAST v5.0.2 5a8b44f within PATRIC (version 3.6.8) for assembly quality assessment. Finally, the assembled contigs were uploaded to the AntiSMASH web platform (http://antismashwebserver.secondarymetabolites.org/) (Blin et al. 2021) for the identification, annotation, and comprehensive analysis of secondary metabolite gene clusters. The identified gene cluster was then used as a reference to design specific primers for the isolation of the BC B-subdomain coding gene.

### Isolation of the BC B-subdomain gene

The isolation of the BC B-subdomain gene from Lac3 involved several steps. First, specific primers were designed using SnapGene software (Table 1) and used to amplify the target region via PCR. Genomic DNA of Lac3 served as the template for the PCR reaction, which was carried out under the following conditions: initial denaturation at 95°C for 3 min; 30 cycles of denaturation at 94°C for 30 s, annealing at 57°C for 30 s, and extension at 72°C for 30 s; followed by a final extension at 72°C for 6 min, using KOD DNA Polymerase (Novagen). The resulting PCR product was purified using the QIAquick Gel Extraction Kit (Qiagen).

**Table 1:** The specific primers of the biotin carboxylase B-subdomain gene

Primers	Primer sequences	Tm [°C]
Primer forward	5’-ATGATGCTGAAATGCATGATGGTT-3’	57
Primer reverse	5’-TTACTTCAATGAAATAAAA TTTATCATCTTTAACAAGAAAC-3’	56

For downstream cloning, a poly-A tail was added to the purified PCR fragment to facilitate binding with the T-overhang ends in the multiple cloning site (MCS) of the pGEM-T Easy cloning vector (Promega). The poly-A tailing reaction was performed by mixing 2× KOD Fx Neo buffer, 2 mM dNTPs, the BC B-subdomain PCR fragment, and Poly-A Mix (Toyobo), followed by incubation at 60°C for 30 min (Mustopa et al. 2022). Ligation of the poly-A–tailed fragments into the pGEM-T Easy vector was carried out at a 1 : 3 ratio by combining 2× rapid ligation buffer, pGEM-T easy cloning vector, and T4 DNA ligase into the BC B-subdomain-poly A mixture, followed by incubation at 4°C for 16 h.

Transformation into *E. coli* DH5α competent cells was performed using the heat shock method (Sambrook and Russell 2001). Transformants were screened via colony PCR using the designed primers (Table 1). The recombinant plasmid was isolated using the Presto Mini Plasmid Isolation Kit (Geneaid), and the presence of the BC B-subdomain gene was confirmed by sequencing.

### 3 D structure prediction and physicochemical analysis

The amino acid sequence of the BC B-subdomain was obtained by translating the gene using SnapGene, followed by *in silico* analysis to predict its physicochemical properties and 3D structure. The ExPASy ProtParam tool (https://www.expasy.org/resources/protparam) was used to assess key parameters such as hydrophobicity, isoelectric point, and molecular weight. Additionally, the Protein-sol sequence solubility webserver (manchester.ac.uk) was used to predict the protein’s overall solubility in water (Niwa et al. 2009). AlphaFold2 was used to predict the 3D structure. The predicted structure, in .pdb format, was analyzed for stability using the SAVES v6.0 web server (Structure Validation Server, https://saves.mbi.ucla.edu/) (DasGupta et al. 2015). YASARA software (Land and Humble 2018) was used to determine the percentage of secondary structure elements, including α helices, β sheets, and coils. Furthermore, CASTp 3.0 software (Tian et al. 2018) was used to analyze potential voids on the protein surface, which may indicate the position of functionally important amino acids.

### Molecular docking analysis

Molecular docking analysis was performed to predict how the BC B-subdomain interacts with its potential ligand, ATP. The 3D structures of both the BC domain from Lac3 reference strain (accession number KLK97304) – hereafter referred to as the BC domain – and the BC B-subdomain were predicted using Alpha-Fold2, along with the ATP ligand. All structures were prepared for docking using BIOVIA Discovery Studio Visualizer v16.1.0.15350. The BC B-subdomain underwent additional validation using the MMPSA method in AMBER 18 (Case et al. 2018) before docking with ATP.

Both the receptor and ligand structures were converted from .pdb to .pdbqt format, and energy minimization was performed using the PyRx Virtual Screening Tool to ensure docking compatibility. Since the ATP binding sites were unknown, a blind docking approach was adopted. AutoDock Vina was used to carry out the simulations, with specific center coordinates and grid box dimensions defined for each receptor. For the BC domain, docking was performed with center points at *x* = –0.8682, *y* = –0.2646, *z* = –0.8610, and a grid box size of 64.2553 Å × 50.7659 Å × 62.5142 Å. For the BC B-subdomain, the center points were *x* = 0.0197, *y* = –10.6789, *z* = 2.4084, and the grid box size was 50.0149 Å × 46.483 Å × 42.9881 Å.

The virtual screening results were analyzed to identify the binding sites on each receptor with the most favorable combination of binding affinity energy, electrostatic interactions (hydrogen bonds), and hydrophobic interactions. Finally, the Protein–Ligand Interaction Profiler (PLIP) web server (https://plip-tool.biotec.tu-dresden.de/plip-web/plip/index) was used to visualize specific chemical bonds between amino acid residues and the ATP ligand within the 3D protein structures.

### Molecular dynamics simulations

The stability of the BC B-subdomain in complex with ATP was assessed using molecular dynamics (MD) simulations performed with AMBER MD 20 (Salomon-Ferrer et al. 2013). The LEaP program was used to set up the systems, applying the General AMBER Force Field (GAFF) for ATP ligands and the ff14SB force field for the protein (Maier et al. 2015). Each system was neutralized with four sodium (Na^+^) cations and solvated in a cubic box (dimensions: 71.755 Å × 71.808 Å × 69.404 Å) using the TIP3P water model (Jorgensen et al. 1983). Particle Mesh Ewald (PME) was employed to manage electrostatic interactions (Essmann et al. 1995), and the SHAKE algorithm was used to constrain hydrogen bond lengths (Ryckaert et al. 1977). A 10 Å cutoff was applied for nonbonded interactions, and a 2 femtosecond (fs) time step was used.

Energy minimization was performed using 10,000 steps of steepest descent followed by 10,000 steps of conjugate gradient to relax the water molecules and eliminate potential steric clashes. The systems were then gradually heated from 0 to 300 K using the canonical ensemble (NVT), followed by a 4 ns equilibration phase under the isothermal-isobaric ensemble (NPT). A 200 ns production MD simulation was subsequently conducted using the Langevin thermostat with a collision frequency of 5 ps (Arwansyah et al. 2022a; Arwansyah et al. 2022b).

Trajectory analyses were performed using CPPTRAJ software (Roe and Cheatham 2013; Mustopa et al. 2023) to evaluate parameters including Root Mean Square Deviation (RMSD), Root Mean Square Fluctuation (RMSF), Solvent-Accessible Surface Area (SASA), and Radius of Gyration (Rg). The conformation of the protein–ligand complex throughout the simulation was visualized using PyMOL software v2.3 (https://pymol.org/2/).

## Results

### Gene cluster analysis

**Figure 1:**
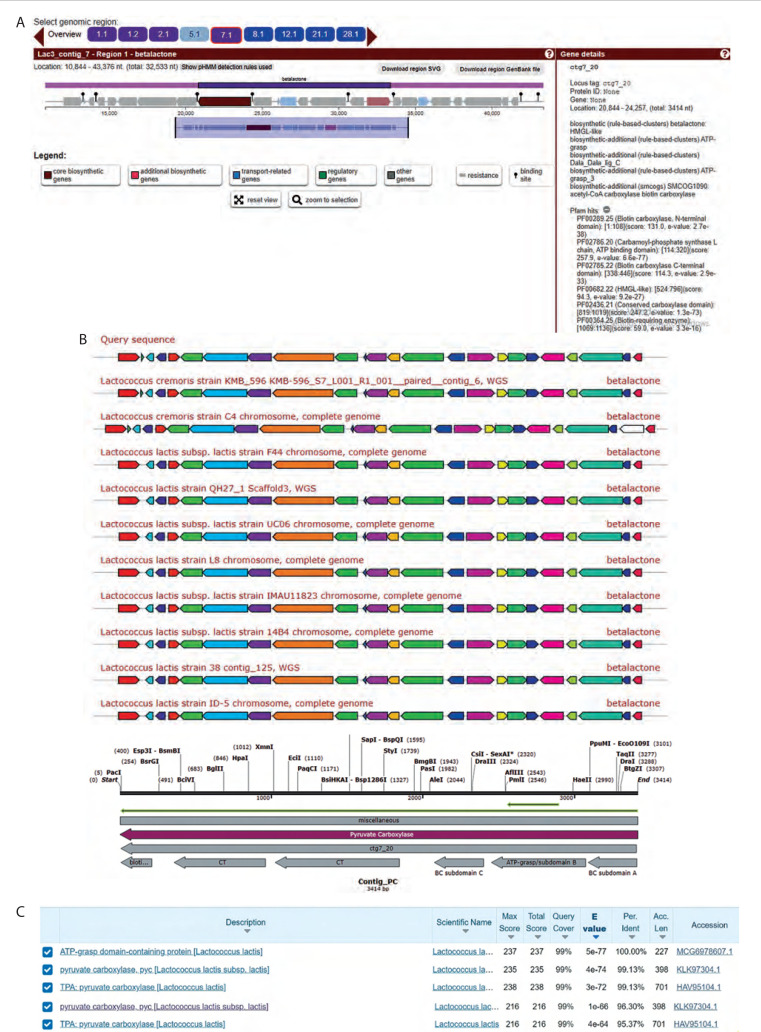
Gene cluster analysis of primary metabolite coding genes in the genome of *L. lactis* subsp. *lactis* (Lac3). **A**) The cluster gene of pyruvate carboxylase (PC) was identified in contig 7.1 using the AntiSMASH analysis of the β-lactone gene cluster. **B**) Protein BLAST analysis of the PC. **C**) Protein BLAST analysis of the biotin carboxylase domain

AntiSMASH gene cluster analysis of the whole genome of Lac3 (accession number NZ_JAGRPZ0100000- 35.1) revealed six clusters of secondary metabolite biosynthetic genes, as shown in Figure 1A. One of these clusters, the NRPS/PKS secondary metabolite β-lactone, was identified on contig 7.1 and exhibited 100% cluster similarity with the Lac3 reference strain (accession number KLK97304). β-lactones are a group of heterocycles characterized by a highly reactive electrophilic region and are known to function as intermediates in the synthesis of various bioactive compounds.

Bioinformatics analysis indicated that the β-lactone gene cluster from Lac3 consisted of biosynthetic core genes, additional biosynthetic genes, transport-related genes, regulatory genes, and other functional elements.

The biosynthetic core genes included both upstream and downstream open reading frames (ORFs). The upstream ORF was identified using PFAM (Protein Family Database) hits and showed similarity to biotin carboxylase (BC) and carbamoyl-phosphate synthase. Protein BLAST analysis revealed 99.13% similarity between this upstream ORF and PC, as shown in Figure 1B. Furthermore, the BC domain within the PC region showed 96.3% similarity with the ATP-grasp domain of Lac3, as illustrated in Figure 1C. The downstream ORF, also identified using PFAM hits, showed similarity to an AMPbinding enzyme. Additional analysis using MiBiG indicated that the downstream ORF shared similarity with acyl-CoA synthetase.

### Isolation of the BC B-subdomain gene

**Figure 2:**
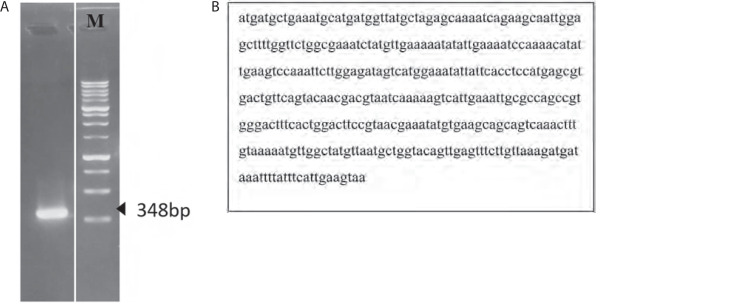
Isolation of the biotin carboxylase (BC) B-subdomain gene from *L. lactis* subsp. *lactis* (Lac3) genome. **A**) Electrophoresis of the BC B-subdomain gene (348 bp). B) Nucleotide sequence of the BC B-subdomain

PCR amplification of the BC B-subdomain gene yielded a fragment of 348 bp (Figure 2A), which was confirmed by sequencing (Figure 2B). Sequence analysis revealed that the BC B-subdomain identified in this study shared similarity with the ATP-grasp region within the BC domain of Lac3 reference strain (accession number KLK97304). In contrast, the full-length BC domain from Lac3 was 1,266 bp in length.

### 3D structure prediction and physicochemical analysis

**Figure 3:**
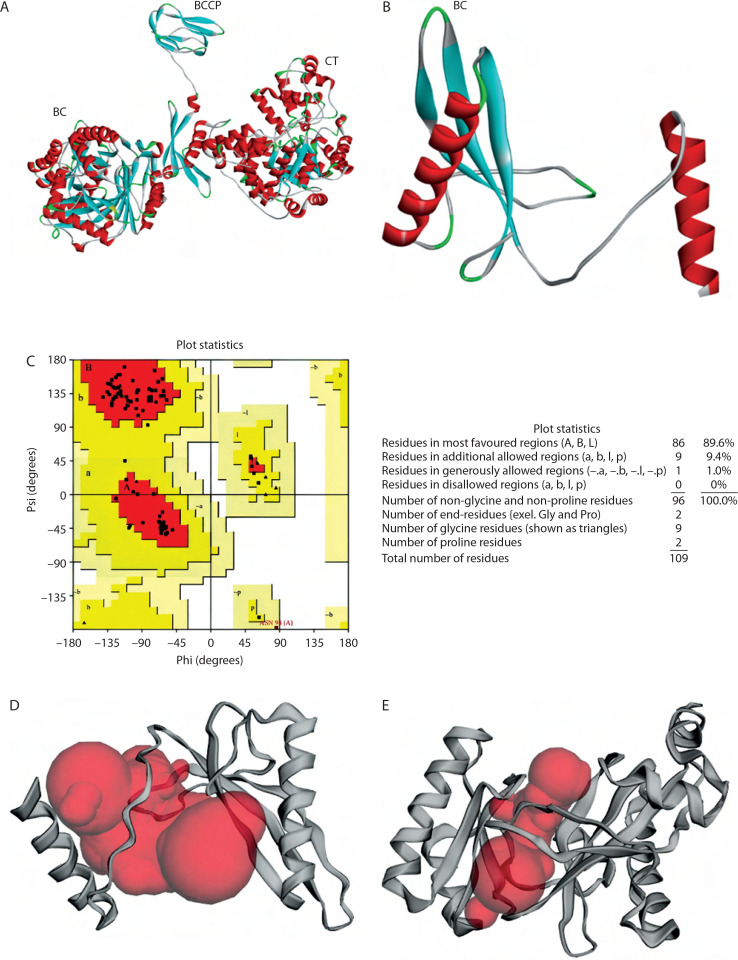
3D structure prediction and physicochemical analysis of proteins. **A**) Protein structure prediction of pyruvate carboxylase (PC). **B**) Protein structure prediction of biotin carboxylase (BC) B-subdomain. **C**) Ramachandran Plot of the BC B-subdomain. **D**) Binding pocket of BC B-subdomain. **E**) Binding pocket of full-length BC domain

Protein structure predictions of PC and the BC B-subdomain were derived from their respective secondary structural components. The PC region in Lac3 showed 98.95% similarity with that of the reference strain (accession number KLK97304). The 3D structure of the PC protein (1,137 amino acids) was predicted using AlphaFold2 and revealed a multidomain architecture comprising three functional domains: BCCP, BC, and CT, as shown in Figure 3A.

The BC B-subdomain was composed of 32.1% α-helix, 37.6% β-strand, 5.5% turn, and 24.8% random coil (Figure 3B). This subdomain in Lac3 exhibited 96.3% similarity with the BC domain of the reference strain (accession number KLK97304), particularly across amino acids 176–284. Protein structure validation for the BC B-subdomain was performed using a Ramachandran plot, which showed that approximately 89.6% of the residues were located in the most favored region (quadrant I), while no residues were present in the disallowed region (quadrant IV) (Figure 3C).

The topographic features of both the BC B-subdomain and the BC domain, including surface protein pockets, are shown in Figures 3D and 3E, respectively. The binding pockets of the BC B-subdomain and the BC domain share several amino acid residues, including Ala183, Glu186, Ala187, Phe191, Ile196, Tyr197, Glu199, Lys200, Tyr201, Ile202, Glu203, Pro205, His207, Ile212, Gly218, Ile220, His222, Leu223, His224, Asp227, Val230, Gln231, Asn234, Lys236, Ile238, Ile240, Phe250, Arg251, Asn252, Cys255, Leu261, Cys262, Gly266, Tyr267, Gly271, Thr272, Val273, Glu274, Leu276, and His284. The area and volume of the BC B-subdomain pocket were calculated to be 486.905 Å^2^ and 339.518 Å^3^, respectively.

Physicochemical analysis was conducted to assess the solubility of the BC B-subdomain. The BC B-subdomain protein showed a pI of 5.82 with a molecular weight of 12,177.87 g/mol. The protein contained 16 negatively charged residues – Asp (4.6%) and Glu (10.1%) and 12 positively charged residues – Lys (4.6%) and Arg (6.4%). The percentages of hydrophobic amino acids Val, Leu, and Ile were 9.2%, 6.4%, and 10.1%, respectively. Aromatic amino acid content included Phe (3.7%), Tyr (3.7%), and Trp (0%). In addition, further confirmation using the Protein-Sol webserver showed a solubility of 0.665.

### Molecular docking analysis

**Figure 4:**
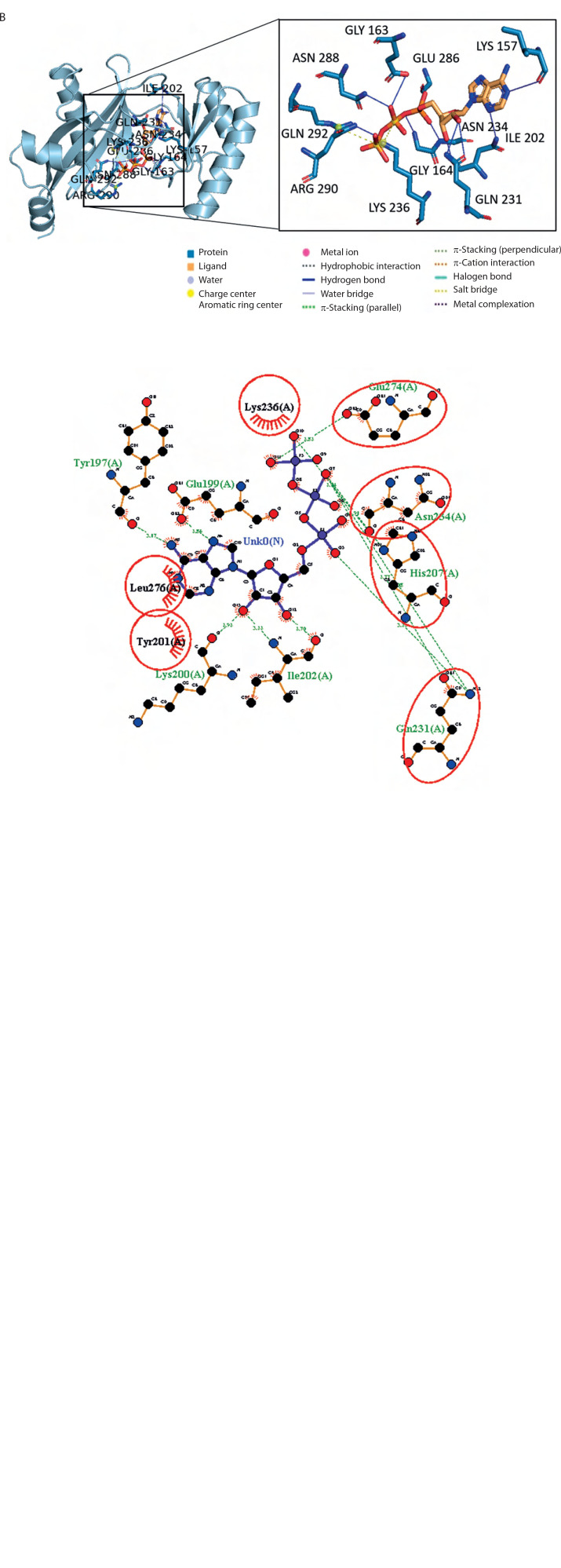
3D and 2D visualization of the binding pocket of the biotin carboxylase (BC) B-subdomain and the BC domain with ATP using molecular docking. **A**) BC B-subdomain of *L. lactis* subsp. *lactis* (Lac3)–ATP complex (–6.1 kcal/mol). **B**) BC domain of *L. lactis* subsp. *lactis* reference strain (accession number KLK97304)–ATP complex (–8.8 kcal/mol)

The molecular binding between the BC B-subdomain and ATP was compared to the binding between the BC domain and ATP using molecular docking analysis. The binding affinity of the BC B-subdomain–ATP complex (–6.1 kcal/mol) was comparable to that of the BC domain–ATP complex (–8.8 kcal/mol), as shown in Figure 4. Both complexes shared key amino acid residues – His207, Gln231, Asn234, and Glu274 – involved in ATP binding, suggesting that the BC B-subdomain may possess ATP-binding activity.

In both complexes, the side chains of His207, Gln231, and Asn274 interacted with the ribose component of ATP. Histidine, a unique amino acid, can function as either an acid or a base. This dual nature makes histidine crucial for various enzymatic reactions. Glutamine and asparagine, with their polar, uncharged amide side chains, facilitate hydrogen bonding and contribute to protein structure and function. Glutamic acid, which has a polar, acidic side chain, can ionize and become negatively charged, enabling its involvement in protein–protein interactions and enzyme catalysis. The hydrogen bond interactions between ATP and both protein domains were generally within the range of 2.5–3.5 Å.

**Figure 5:**
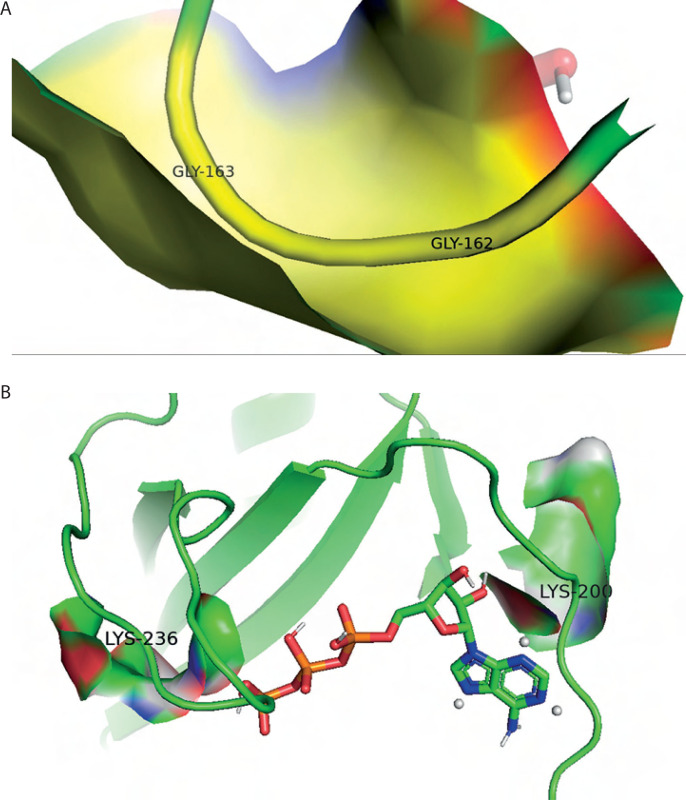
Key amino acids of P-loop binding. **A**) Gly-loop interactions between the biotin carboxylase (BC) domain (accession number KLK97304) with ATP. **B**) Lysine hydrophobic and ionic interactions between the BC B-subdomain with ATP

Additionally, hydrophobic interactions were identified in the BC B-subdomain, involving residues such as Tyr201 and Leu276 (Table 2). Ionic interactions with the phosphate groups of ATP were also observed in both the BC B-subdomain (His207 and Lys236) and the BC domain (Lys236 and Arg290), as shown in Table 3. Furthermore, glycine-loop interactions (Gly162 and Gly163) were identified in the BC domain–ATP complex (Figure 5A). Although no glycine-loop interaction was detected in the BC B-subdomain, hydrophobic and ionic interactions involving Lys236 and Lys200 were observed in the loop region. Both glycine and lysine are critical residues in P-loop binding motifs.

**Table 2:** Hydrogen bonds and hydrophobic interactions between the biotin carboxylase (BC) B-subdomain and the BC domain with ATP

No	Complex	Hydrogen bond residue	Hydrogen bond distance [Å]	Hydrophobic interaction
1	BC B-subdomain with ATP	Gln231	3.11 3.06 2.11	Tyr201 Lys236 Leu276
		His207	2.91	
		Glu274	2.63	
		ASN234	2.90	
		Ile202	2.69 3.22	
		Lys200	2.91	
		Tyr197	3.11	
		Glu199	2.56	
2	BC domain with ATP	Gln231	2.98 3.25	Tyr201 Met167 Met155 Leu276 Gly162
		Glu274	2.92 2.98	
		Asn234	2.70	
		Asn288	3.10 3.21 2.81	
		Lys236	3.07	
		Arg290	3.13 3.01	
		His207	2.94	
		Glu286	3.08	
		Gly164	2.90	
		Gly163	2.90	
		Gln292	2.81	

**Table 3:** Ionic interactions (salt bridges) between ATP with the biotin carboxylase (BC) B-subdomain and the BC domain

Domain	Residue	AA	Distance [Å]	Ligand group
BC B-subdomain	199A	GLU	5.46	Tertamine
	207A	HIS	4.72	Phosphate
	207A	HIS	4.52	Phosphate
	236A	LYS	5.38	Phosphate
BC	236A	LYS	4.49	Phosphate
	236A	LYS	5.44	Phosphate
	290A	ARG	4.58	Phosphate

### Molecular dynamics simulations

The structural stability of the docked complex was validated by performing all-atom MD simulations using the TIP3P water model system. Several validation parameters – including RMSD, RMSF, Rg, and SASA – were assessed for each complex.

**Figure 6:**
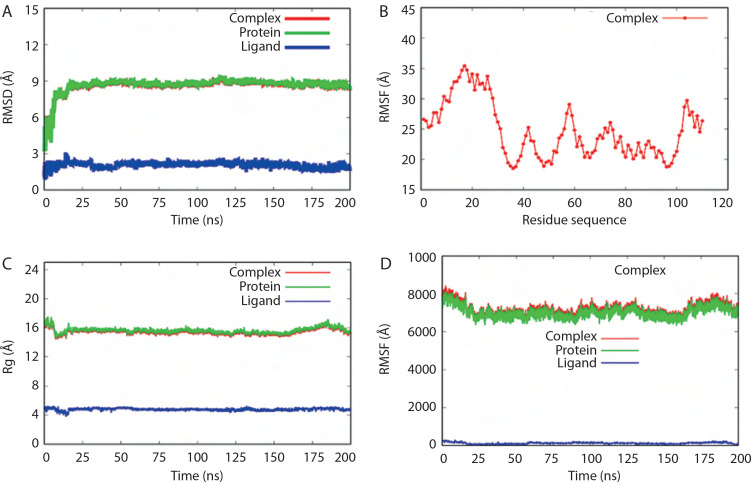
MD validation metrics. **A**) RMSD value of the complexes. **B**) RMSF descriptors. **C**) Rg profiles. **D**) Protein surface area measured by SASA analysis. Complex, protein, and ligand were denoted by biotin carboxylase (BC) B-subdomain in complex with ATP, BC B-subdomain, and ATP, respectively

RMSD values over the simulation time for the BC B-subdomain, ATP ligand, and the BC B-subdomain–ATP complex are depicted in Figure 6A. No significant fluctuations were observed, suggesting that all models remained stable throughout the simulation. The RMSF profile, which reflects the flexibility of individual amino acid residues within the BC B-subdomain– ATP complex, is shown in Figure 6B. The trend suggested a generally flexible structure, supporting the ability of ATP to bind effectively to the BC B-subdomain.

The Rg values of the BC B-subdomain, ATP, and the complex are shown in Figure 6C. These values, associated with molecular compactness, did not change significantly during the simulation, indicating that each model maintained a compact structure. SASA values for the BC B-subdomain, ATP, and their complex are presented in Figure 6D. The BC B-subdomain and the complex exhibited greater surface areas than the ATP ligand alone, which enhances the protein’s potential to form interactions.

**Figure 7:**
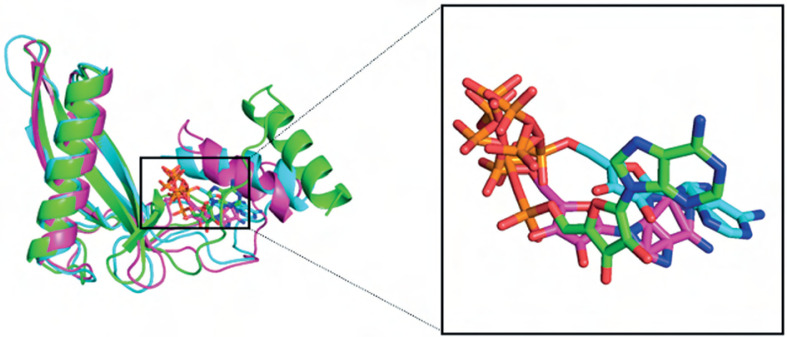
Superimposes of protein–ligand complex. Molecular docking (green), molecular dynamic simulations at 100 ns (cyan), and 200 ns (magenta). Protein and ligand were denoted by cartoon and stick models, respectively

Overall, the BC B-subdomain–ATP complex demonstrated structural stability and flexibility, as illustrated in Figure 7.

## Discussion

PC, a large enzyme found in many organisms, catalyzes the conversion of pyruvate to oxaloacetate in two steps. First, it uses ATP and bicarbonate to carboxylate its biotin cofactor, and then transfers the carboxyl group to pyruvate. PC consists of three main parts: BC, BCCP, and CT domains (Sueda et al. 2004). Although the BC domain shares some structural similarity with eukaryotic kinases, it possesses unique features that make it a promising target for the development of antibacterial drugs capable of selectively inhibiting bacterial PC without affecting the host (Miller et al. 2009).

In Lac3, the BC domain shares 96.3% similarity with that of Lac3 strains isolated from nondairy sources. The BC domain in the reference isolate is 1,194 bp in length and includes the ATP-grasp domain around the 585 bp region (Cavanagh et al. 2015).

The BC domain comprises three subdomains – A, B, and C – which are part of the biotin-dependent carboxylase enzyme family. The ATP-binding site is located within the ATP-grasp fold near the hinged lid region, known as the BC B-subdomain, which envelops the ATP molecule. The BC B-subdomain consists of two α-helices and three β-sheets, and includes a glycine-loop (residues 160–166) that plays a critical role in ATP binding. During catalysis, the BC B-subdomain undergoes dimerization and closes the active site (Thoden et al. 2000). Kinetic studies have shown that ATP binding is enhanced in the presence of the biotin cofactor, indicating a synergistic relationship between ATP and biotin during enzymatic activity (Mochalkin et al. 2008).

The biotin carboxylase B-subdomain undergoes a significant conformational change upon ATP binding, which is essential for its catalytic function. This conformational shift optimizes substrate positioning, facilitating the activation of bicarbonate and its subsequent transfer to biotin. While the specific details of active-site residue interactions in *L. lactis* may require further investigation, the broader context of the ATP-grasp enzyme superfamily provides valuable insight (Lopez-Alonso et al. 2022). Members of this superfamily share a conserved ATP-grasp fold that enables ATP-dependent carboxylation reactions. Previous studies have shown that the ATP-binding site in ATP-grasp enzymes is distinct from that of other ATP-binding proteins, based on observations using strong transition state analogs. This distinction supports the rationale that the ATP-grasp binding site may serve as a viable target for drug design (Fawaz et al. 2011).

The initial characterization of the BC B-subdomain within the genome of Lac3 was facilitated by the Anti- SMASH webserver. This bioinformatic tool integrates a sophisticated rule-based system – curated and manually validated – to delineate biosynthetic core regions within genomic data. Its analytical strength lies in its use of profile hidden Markov models (pHMMs) derived from multiple databases, including PFAM, TIGRGAMS, SMART, BAGEL, as well as specialized modules for nonribosomal peptide synthetase (NRPS), polyketide synthase (PKS), and ribosomally synthesized and posttranslationally modified peptide (RiPP) functional annotations (Blin et al. 2021).

To investigate the structural organization of the identified BC B-subdomain, its secondary structure was predicted using a dual approach involving both PSI-BLAST and PSI-PRED algorithms. This combined method provided probability scores for three major conformational states: coil, helix, and strand. The predicted secondary structure served as the basis for evaluating the stability of the BC B-subdomain using a Ramachandran diagram, which assesses protein structure quality by examining the distribution of non-glycine amino acid residues. A well-folded protein typically has over 90% of its residues in allowed regions and fewer than 20% in quadrant IV (Carugo and Djinovic-Carugo 2013). The Ramachandran diagram essentially represents a two-dimensional plot of phi (Ф) and psi (ψ) backbone torsion angle pairs. The precise structural conformation of the protein is further refined by analyzing contact plots between individual atoms (DasGupta et al. 2015). This multi-faceted computational approach provided a comprehensive initial assessment of the BC B-subdomain’s genomic context, predicted secondary structure, and potential stability.

Physicochemical analysis of the BC B-subdomain was performed using the ProtParam tool on the ExPASy webserver, based on its isoelectric point (pI) and amino acid composition. A pI in the range of 5–7 typically indicates high solubility. Higher proportions of negatively charged amino acid residues are generally associated with increased solubility, whereas aromatic residues and hydrophobic interactions have minimal impact on protein aggregation. Protein solubility was further assessed using the Protein-Sol webserver, which calculates the population average solubility (PopAvrSol) value. A PopAvrSol value greater than 0.45 suggests high solubility (Niwa et al. 2009).

At its isoelectric point, a protein exhibits reduced electrostatic repulsion and minimal solubility, which can promote aggregation. Below the pI, proteins carry a net positive charge, attracting negatively charged molecules; above the pI, they are negatively charged, attracting positively charged molecules. The pI also influences protein stability by modulating ionic interactions and hydrogen bonding. It is determined by the protein’s amino acid composition and the ionization states of side chains, which vary with pH.

To gain further insight into the functional characteristics of the BC B-subdomain and the reference BC domain, we analyzed their physicochemical features in relation to their spatial organization – particularly amino acid residues located in surface voids and at functionally critical positions. For this purpose, we used the Computed Atlas of Surface Topography of Proteins (CASTp) webserver. CASTp provides a suite of computational tools for identifying, characterizing, and quantifying geometric and topological features on protein surfaces. It is particularly effective at mapping surface pockets, internal cavities, and channels that are often essential for protein function, including ligand binding and substrate accessibility.

CASTp also provides precise calculations of the volume and surface area of these features, as well as the dimensions of openings, using two analytical models: the solvent-accessible surface (Richards’ surface) and the molecular surface (Connolly’s surface), providing a comprehensive understanding of the protein’s surface landscape and its potential implications for molecular interactions (Tian et al. 2018). This detailed characterization of the surface topography and internal architecture is crucial for inferring the potential binding sites and functional mechanisms of the BC B-subdomain and the BC domain.

To gain insight into the ligand-binding capabilities of our target proteins, we employed molecular docking – a computational technique designed to predict the preferred orientation of a ligand within the active site of a receptor molecule. The fundamental principle of molecular docking involves using energy-based search algorithms to generate multiple potential conformations (poses) of the ligand within the designated binding pocket. These poses are then evaluated and ranked using a scoring function that estimates the binding affinity (Du et al. 2016). Essentially, docking simulates the ligand-binding process, beginning with information about the protein’s structure and predicting the most energetically favorable binding mode.

To define the search space for the ligand, grid boxes are typically constructed around known or predicted binding sites on the protein, accounting for the ligand’s translational, rotational, and torsional degrees of freedom (Morris et al. 2008). However, in cases where structural information about the binding site is unavailable – as with the BC domain of the reference strain Lac3 (accession number KLK97304) – a more extensive approach is required. In such scenarios, a “blind docking” procedure is used, wherein the entire protein surface is explored as a potential binding region. AutoDock Vina is well suited for this methodology, as it enables whole-protein docking by employing a sufficiently large grid box to encompass the entire molecular structure (Muscat et al. 2020). This approach allows for the identification of potential binding pockets even in the absence of prior structural knowledge.

The interaction between the BC B-subdomain and the BC domain with ATP involves a complex interplay of electrostatic, hydrophobic, and hydrogen bonding forces. Among these, hydrogen bonds are particularly important. These bonds occur between electronegative atoms – such as oxygen, nitrogen, or fluorine – and a hydrogen atom covalently bonded to an electronegative donor (Arwansyah et al. 2014). A hydrogen bond is formed when this hydrogen shares its electron with another electronegative acceptor atom. The strength of the ligand–receptor interaction is often influenced by the distance between the ligand and surrounding amino acid residues, with closer proximity typically indicating stronger and more stable binding.

Hydrogen bonds are essential for protein structure and function. They stabilize secondary structures such as α-helices and β-sheets and are vital in molecular recognition, guiding the interactions between proteins and their ligands. Efficient hydrogen bond formation is critical for proper protein folding, structural stability, and selective ligand binding (Głowacki et al. 2013).

In addition to hydrogen bonds, the interaction between ATP and both the BC domain and BC B-subdomain also involves hydrophobic forces. Hydrophobic amino acid residues tend to cluster in the protein’s interior, away from the aqueous environment. These interactions – driven by van der Waals forces and the hydrophobic effect – contribute to the stability of the ligand– receptor complex by minimizing Gibbs free energy (Δ*G*) (Zaelani et al. 2021). A negative ΔG value reflects a spontaneous and energetically favorable interaction, with a more negative ∆*G* indicating a stronger and more stable complex. This thermodynamic principle underlies ligand–receptor binding, where a final state characterized by lower free energy is both stable and biologically relevant (Agu et al. 2023).

Ionic interactions with amino acid residues of the phosphate group were identified in both the BC B-subdomain and the BC domain. The phosphate group plays a critical role in ATP binding and enzymatic function within ATP-grasp enzymes. Disruption of this interaction can significantly impair enzyme activity. Due to its distinct chemical structure, the phosphate group presents an attractive target for drug development, enabling the design of molecules that specifically bind to it while minimizing off-target effects on other cellular components (Pederick et al. 2020). The difficulty lies in making ATP-like inhibitors that lack phosphate groups. These groups, while crucial for strong binding between ATP and ATPases, also make the molecules less readily absorbed by the body (low bioavailability) and more prone to breaking down (poor stability). The alternative way to block the activity of ATPases is to create molecules that compete with ATP for the binding site. This method has worked well for designing drugs that target protein kinases, which depend on the structure of the nucleotide-binding site (Chčne 2002). The phosphate group of ATP forms multiple interactions with the nucleotide binding site of ATPase, significantly contributing to the binding energy of the nucleotide.

Additionally, ionic interactions were observed between the amino acid Glu199 and the tertamine group within the BC B-subdomain. However, there is currently no scientific consensus regarding tertamine as a natural ligand for ATP-grasp enzymes. Further studies are needed to explore the nature and significance of tertamine interactions in this enzyme family.

The glycine loop acts like a flexible handshake with ATP. Its small size and the nearby amino acids with positive charges allow it to grip the negatively charged phosphates of ATP securely. Glycine has a hydrogen and hydrocarbon side chain, also has a quirk that helps it interact with other molecules. While the positive and negative groups do not directly link glycine molecules together (covalent bonding), they do create a special attraction (Li et al. 2007). These groups act like tiny magnets, allowing glycine to form hydrogen bonds with itself and other glycine molecules. These bonds are key to shaping the structure of glycine and how it interacts with its surroundings (Sirois et al. 1997).

Walker-A sequence within the P-loop (phosphate-loop), specifically GxxGxGK[T/S], binds to phosphate group on molecules called phosphorylated ribonucleosides and facilitates phosphate transfer (Romero et al. 2018). The P-loop is flanked by a β-strand and an α-helix, forming a structural motif that stabilizes phosphate interactions. The flexible glycine residues in the P-loop, along with a crucial lysine, create a hollow structure that accommodates the phosphate group. The phosphate’s negative charge is attracted to the lysine’s positive charge, anchoring the phosphate securely in place (Laurino et al. 2016).

Molecular dynamics simulations, based on the computational calculation of Newtonian equations of motion and the application of various force fields, offer a powerful and cost-effective alternative to experimental methods for predicting and analyzing the behavior of atoms and molecules (Roe and Cheatham 2013). In this study, several MD simulation parameters – RMSD, RMSF, Rg, and SASA – were employed to assess the stability and dynamics of the BC B-subdomain, ATP ligand, and their complex. RMSD measures the average atomic displacement over time, comparing each simulation frame to a reference structure and providing insight into overall structural stability (Roe and Cheatham 2013). Rootmean-square fluctuation (RMSF) assesses the average movement of individual atoms relative to their mean position, highlighting the flexibility of amino acid residues throughout the simulation. Typically, as a molecular system approaches equilibrium, RMSD and RMSF values stabilize, forming a plateau that indicates structural consistency. Rg represents the root-weighted average distance of atomic masses from the protein’s center of mass, reflecting its overall compactness. SASA quantifies the protein surface exposed to the solvent, offering insight into its interaction potential (Bonet et al. 2021). Overall, the BC B-subdomain, ATP ligand, and their complex exhibited consistent structural behavior. The BC B-subdomain–ATP complex, in particular, demonstrated both stability and flexibility, with no significant fluctuations observed throughout the simulation period.

## Conclusion

This study successfully isolated the BC B-subdomain from Lac3 (accession number NZ_JAGRPZ0100000-35.1). ATP-binding sites were predicted in both the BC B-subdomain and the BC domain of the *L. lactis* reference strain (accession number KLK97304). Molecular docking analysis revealed that both the BC B-subdomain and BC domain shared key hydrogen-bonding residues – His207, Gln231, Asn234, and Glu274 – and exhibited ionic interactions with the phosphate groups of ATP. The BC B-subdomain utilized distinct loop region residues (Lys236 and Lys200) for hydrophobic and ionic interactions, whereas the BC domain employed the glycine loop (Gly162 and Gly163). The BC B-subdomain–ATP complex, which lacked the glycine loop, was slightly less energetically favorable (–6.1 kcal/mol) than the BC domain–ATP complex (–8.8 kcal/mol). Nonetheless, the BC B-subdomain retained ATP-binding capability comparable to that of the full BC domain. Furthermore, molecular dynamics simulations confirmed the structural stability and flexibility of the BC B-subdomain–ATP complex. These findings suggest that the BC B-subdomain represents a promising target for fragmentbased drug discovery. However, further investigations – such as comprehensive molecular profiling of nucleotidebinding proteins including ATP-grasp enzymes, ATPases, and kinases – are needed to support the development of innovative therapeutic strategies.
